# Sleep alterations in treatment-resistant depression patients undergoing ketamine treatment

**DOI:** 10.1007/s43440-024-00641-1

**Published:** 2024-08-29

**Authors:** Aleksander Kwaśny, Wiesław Jerzy Cubała, Adam Włodarczyk, Krzysztof Pastuszak

**Affiliations:** 1 Department of Psychiatry, Faculty of Medicine, Medical University of Gdańsk, Gdańsk, 80-214 Poland; 2 Department of Algorithms and System Modeling, Gdansk University of Technology, Gdańsk, Poland; 3 Laboratory of Translational Oncology, Intercollegiate Faculty of Biotechnology, University of Gdańsk and Medical University of Gdańsk, Gdańsk, Poland; 4 Center of Biostatistics and Bioinformatics, Medical University of Gdańsk, Gdańsk, Poland

**Keywords:** Sleep, Depression, Major depressive disorder, Treatment-resistant depression, Ketamine

## Abstract

**Background:**

This study examines self-reported sleep alterations in treatment-resistant depression (TRD) inpatients following intravenous ketamine administration.

**Methods:**

This is a post-hoc analysis of a naturalistic observational study, which enrolled 28 inpatients with treatment-resistant major depressive disorder and analyzed self-reported sleep changes (items 1–4; ‘insomnia’, ‘nighttime restlessness’, ‘early morning waking’, ‘hypersomnia’) in Inventory of Depressive Symptomatology 30-item (IDS SR-30) in responders and non-responders stratified per Montgomery-Åsberg Depression Rating Scale (MADRS) during short-term ketamine treatment.

**Results:**

Responders, as well as non-responders, did not experience significant changes in IDS SR-30 sleep items (‘insomnia’, ‘nighttime restlessness’, ‘early morning waking’, ‘hypersomnia’) (p’s > 0.05) at 7-day follow-up after eight intravenous ketamine infusions as compared to baseline.

**Conclusion:**

Neither responders, nor non-responders reported any significant alterations in sleep patterns during ketamine infusions. These findings are not in line with current literature, as so far modest improvements in sleep during ketamine treatment have been reported. Results should be interpreted with caution, primarily due to the small sample size.

## Introduction

Major depressive disorder (MDD) is a widespread psychiatric entity with around 350 million individuals affected around the world and represents the second leading cause of disease burden worldwide [1]. Treatment-resistant depression (TRD) is a major challenge for the psychopharmacology of affective disorders. Treatment resistance is commonly referred to as two failures of antidepressant trials in an adequate dose for an adequate time, however, there is no consensus on the definition [2].

Sleep disturbances are often reported by patients with major depression. Over 90% of patients report some form of impaired sleep [3] with insomnia being one of the most common residual symptoms [4]. Insomnia is frequent in the TRD population and serves as a predictor for developing one [5]. Similar to factors such as hopelessness or certain affective temperaments, mounting evidence indicates that poor sleep quality is a significant risk factor for suicide, independent of depression severity [6–8]. Compared to non-suicidal individuals, those at risk for suicide show higher mean concentrations of inflammatory mediators in both the periphery and brain. Evidence suggests that neuroinflammation is associated with dysregulation of the kynurenine pathway, leading to an imbalance of neuroactive metabolites in suicidal individuals with TRD [9].

Ketamine is a racemic mixture of esketamine and arketamine and shows a strong binding affinity for the phencyclidine site on the N-methyl-D-aspartate receptor (NMDAR). By antagonizing NMDARs on γ-aminobutyric acid (GABA) interneurons, ketamine inhibits their tonic firing, leading to a surge in glutamate. This increase in glutamate activates ionotropic α-amino-3-hydroxy-5-methyl-4-isoxazolepropionic acid receptors (AMPAR), which enhances brain-derived neurotrophic factor (BDNF)-TrkB-ERK and PI3-AKT-mTOR pathways [10]. Thus, through its modulation of glutamatergic neurotransmission, ketamine is associated with rapid reductions in depressive symptoms and suicidal thoughts [11, 12]. Lately, significant progress has been made in the use of rapid-acting antidepressant medications to treat individuals with TRD. Notably, the nasal spray form of ketamine’s enantiomer i.e., esketamine, when combined with approved antidepressants for TRD, has shown effectiveness in quickly alleviating depressive symptoms in individuals diagnosed with MDD [13]. Additionally, evidence indicates that esketamine nasal spray rapidly and effectively reduces depressive symptoms in patients with major depressive disorder who have severe illness and active suicidal ideation with intent [14, 15]. These findings offer strong evidence supporting the use of ketamine due to its rapid-acting antidepressant properties in MDD. There is evidence this effect may be in part mediated by circadian regulation and sleep improvement [16–18].

Recently, the focus has been put on researching and treating symptom domains as presented in Research Domain Criteria (RDoC), among which are circadian rhythms and sleep-wakefulness domains [19].

Hence, the objective of this post-hoc analysis within the MDD TRD sample is to explore the correlation between ketamine administration and alterations in sleep patterns, as well as the potential influence of sleep on mediating antidepressant and anti-suicidal effects. Consistent with existing literature, it may be hypothesized that ketamine could enhance overall sleep quality.

## Methods

### Patients

This is a retrospective analysis of the study population, which consisted of individuals included in a naturalistic observational registry protocol for intravenous (IV) ketamine treatment in TRD. A comprehensive description of the study population and methodology can be found elsewhere [20]. Briefly, the diagnostic criteria followed the guidelines outlined in the Diagnostic and Statistical Manual of Mental Disorders (DSM-5) and focused on individuals with TRD, which is characterized by an inadequate response to two or more antidepressant medications prescribed at appropriate dosages and for an adequate duration. This study was conducted at the Department of Psychiatry at the Medical University of Gdańsk (Poland) and recruited adult inpatients who presented with TRD and were determined to be suitable for short-term IV ketamine treatment. A total of 41 patients were initially enrolled in the study. However, 13 patients were subsequently excluded from further analysis due to a diagnosis of bipolar disorder, resulting in a final cohort of 28 patients. The study was registered on ClinicalTrials.gov under the identifier NCT04226963, received approval from the Independent Bioethics Committee for Scientific Research at the Medical University of Gdańsk, Poland (approval code NKBBN/172–674/2019) and the investigation was carried out by the latest version of the Declaration of Helsinki. Patients provided written informed consent to participate in the study and for the collection of their data.

### Study design

The study adopted an observational registry for safety and tolerability design, in which participants received a total of eight IV ketamine infusions, twice per week, over four weeks as an adjunctive treatment. Patients were subsequently followed up for one week after their final infusion. Ketamine was administered via IV delivery at a 0.5 mg/kg dose, a process that took approximately 40 min.

Throughout the study, rigorous safety and tolerability monitoring occurred continuously, concurrently with assessments conducted for the registry.

### Psychometric measures

Depressive symptoms were assessed using the Inventory of Depressive Symptomatology Self-Report 30 (IDS-SR 30) [21]. This self-report questionnaire provides a comprehensive evaluation of a wide range of depressive symptoms and consists of thirty questions rated on a scale from 0 to 3, with higher scores indicating greater severity of the symptom. The total score is calculated from 28 questions, as two questions should remain unanswered (i.e., only one question is answered for increased or decreased appetite and for weight gain or loss). The total score ranges from 0 to 84 points. Participants rate each item based on the severity of symptoms experienced in the past 7 days including four items concerning sleep disruption (items 1–4; ‘insomnia’, ‘nighttime restlessness’, ‘early morning waking’, ‘hypersomnia’). Participants were asked to rate the severity of each symptom they experienced. Patients were then categorized into two groups: responders and non-responders, based on their scores on the Montgomery-Åsberg Depression Rating Scale (MADRS) [22], the gold standard clinician rating scale for depression [23]. All assessments were performed weekly, with a 7-day follow-up conducted after the 8th infusion.

In this study, a response was defined as a reduction of 50% or more in the MADRS score from the baseline measurement. The classification of participants into responders and non-responders was determined based on their MADRS scores at the seventh infusion.

### Statistical analysis

Statistical analysis was conducted using R (version 4.3.2) software. The distribution of numerical variables was assessed using the Shapiro test. Since none of the considered variables followed a normal distribution, the paired Wilcoxon test was used for pairwise comparisons. *p* < 0.05 was considered significant. No data imputation was used.

## Results

### Baseline characteristics

The demographic and clinical characteristics of the participants are detailed in Table 1. According to the MADRS scores obtained at baseline and following the 7th infusion, the 28 patients were classified into two groups: responders (*n* = 6) and non-responders (*n* = 22).

**Table 1:** Demographic and clinical variables for treatment-resistant major depressive disorder subjects in short-term intravenous ketamine treatment as stratified to responders and non-responders defined as a reduction of 50% or more in the Montgomery-Åsberg Depression Rating Scale (MADRS) score between baseline and 7th infusion

Variables	Responders^1^ (*n* = 6)	Non-responders^2^ (*n* = 22)
**Age**		
Mean (SD)	40,2 (11.2)	51.5 (14.0)
**BMI**		
Mean (SD)	26.3 (6.4)	27.6 (4.9)
**Sex**		
Female	3 (50%)	13 (59.1%)
Male	3 (50%)	9 (40.9%)
**Education**		
Elementary	2 (33.3%)	0 (0%)
Vocational	1 (16.7%)	2 (9.1%)
Secondary	1 (16.7%)	9 (40.9%)
Higher	2 (33.3%)	11 (50%)
**Employment status**		
Unemployed	2 (33.3%)	4 (18.2%)
Pensioner	0 (0%)	10 (45.5%)
Retirement	1 (16.7%)	4 (18.2%)
Employed	3 (50%)	3 (13.6%)
Study	0 (0%)	1 (4.5%)
**Marital status**		
Single	1 (16.7%)	5 (22.7%)
Informal relationship	1 (16.7%)	1 (4.5%)
Married	3 (50%)	11 (50%)
Divorced	1 (16.7%)	3 (13.6%)
Widowed	0 (0%)	2 (9.1%)
**Concomitant meds**		
TCA	1 (16.7%)	3 (13.6%)
SSRI	2 (33.3%)	14 (63.6%)
SNRI	2 (33.3%)	3 (13.6%)
Other*	3 (50%)	9 (40.9%)
Antipsychotics	0 (0%)	7 (31.8%)
Mood stabilizers	2 (33.3%)	8 (36.4%)
**IDS-SR 30**		
Mean (SD)	46.7 (9.5)	47.7 (12.5)
Median	47.5	49
95%CI	(36.7;56.7)	(42.2;53.3)

^1^ Patients diagnosed with major depressive disorder who exhibited a 50% or greater reduction in Montgomery–Åsberg Depression Rating Scale (MADRS) scores from baseline to the 7th infusion during observational registry for safety and tolerability, and who were admitted as inpatients at the Department of Psychiatry, Medical University of Gdańsk (Poland)

^2^ Patients diagnosed with major depressive disorder who exhibited less than 50% reduction in Montgomery–Åsberg Depression Rating Scale (MADRS) scores from baseline to the 7th infusion during observational registry for safety and tolerability, and who were admitted as inpatients at the Department of Psychiatry, Medical University of Gdańsk (Poland)

* - mirtazapine, mianserin, trazodone, bupropion, vortioxetine;

BMI – body mass index; 95% CI – 95% confidence interval; IDS SR-30 – Inventory of Depressive Symptomatology Self-Report 30 item; IQR – interquartile range; MDD – major depressive disorder; SD – standard deviation; SSRI - selective serotonin reuptake inhibitors; SNRI – serotonin and noradrenaline reuptake inhibitors; TCA - tricyclic antidepressants

This categorization reflects severe major depressive episode symptomatology in subjects with TRD who were treated with standard-of-care treatment, including combined therapy with antidepressants, antipsychotics, and mood stabilizers.

### Sleep changes in IDS SR-30

No significant changes were observed in the responders’ group in insomnia (*p* = 0.089, median 2 vs. 0.5), nighttime restlessness (*p* = 0.3222, median 2 vs. 0.5), early-morning waking (*p* = 0.8501, median 1.5 vs. 0.5), and hypersomnia (*p* = 0.0947, median 1 vs. 0), however the limited size of the responder group (*n* = 6) should be noted.

**Fig. 1:**
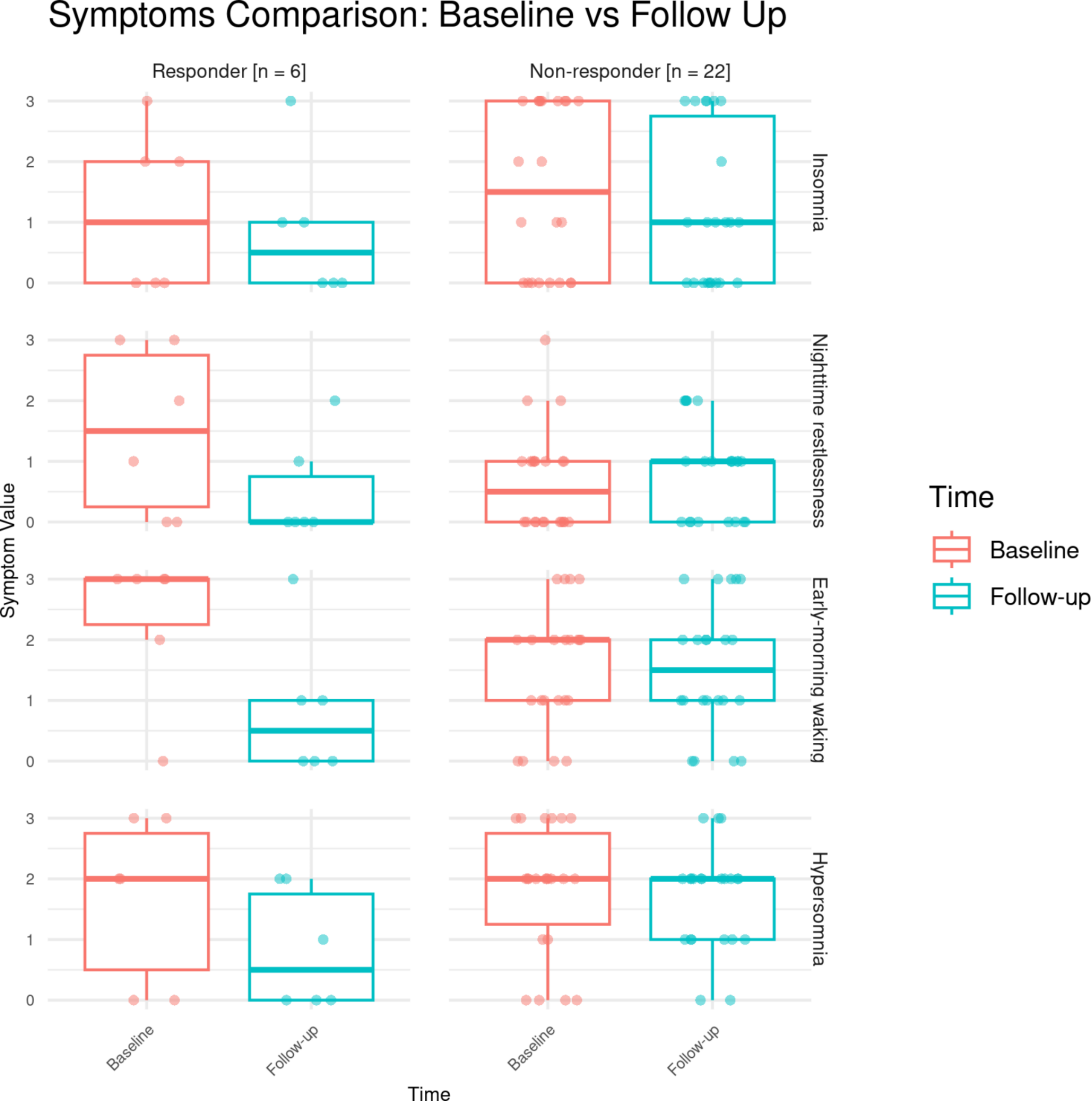
Comparison of sleep-related items according to Inventory of Depressive Symptomatology Self-Report (IDS SR-30) between baseline and 7-day follow-up after 8th ketamine infusion in responders^1^ and non-responders^2^. Boxes contain values between first and third quartiles. Whiskers highlight extreme values within at most 1.5 IQR from Q1 or Q3. Each patient is marked by a separate dot ^1^ Patients diagnosed with major depressive disorder who demonstrated a 50% or greater reduction (*n* = 6, responders) in Montgomery–Åsberg Depression Rating Scale (MADRS) scores from baseline to the 7th ketamine infusion during observational registry for safety and tolerability, and who were admitted as inpatients at the Department of Psychiatry, Medical University of Gdańsk (Poland) ^2^ Patients diagnosed with major depressive disorder who experienced less than 50% reduction (*n* = 22, non-responders) in Montgomery–Åsberg Depression Rating Scale (MADRS) scores from baseline to the 7th ketamine infusion during observational registry for safety and tolerability, and who were admitted as inpatients at the Department of Psychiatry, Medical University of Gdańsk (Poland) Q – quartile IQR – interquartile range

Similar results were reported in the non-responders’ group in insomnia (*p* = 1, median 2 vs. 1.5), nighttime restlessness (*p* = 0.7329, median 2 vs. 2), early-morning waking (*p* = 0.1838, median 1.5 vs. 1), and hypersomnia (*p* = 0.2669, median 1 vs. 1). The distributions of sleep-related IDS-SR 30 items are presented in Fig. 1.

Overall, ketamine infusions did not produce any significant alterations in sleep patterns throughout eight infusions. The detailed results of the analysis are presented in Table 2.

**Table 2:** Comparison of sleep-related items according to inventory of depressive symptomatology self-report (IDS SR-30) between baseline and 7-day follow-up after 8th ketamine infusion in responders^1^ and non-responders^2^

IDS SR-30 item	Responders^1^				Non-responders^2^			
	Baseline	Follow up	Z	*p*	Baseline	Follow up	Z	*p*
Insomnia	2 (1–3)	0.5 (0–1)	1.701	0.089 ^b^	2 (1–3)	1.5 (1–2)	0	1 ^b^
Nighttime restlessness	2 (1–3)	0.5 (0–1.75)	0.990	0.3222 ^b^	2 (1–3)	2 (1–2)	0.341	0.7329 ^b^
Early morning waking	1.5 (0–3)	0.5 (0–1)	0.189	0.8501 ^b^	1.5 (0–3)	1 (0–2.75)	1.329	0.1838 ^b^
Hypersomnia	1 (0–1)	0 (0–0.75)	1.671	0.0947 ^b^	1 (0–1)	1 (0–1)	1.110	0.2669 ^b^

IDS SR-30 – Inventory of Depressive Symptomatology Self-Report 30 item

^1^ Patients diagnosed with major depressive disorder who demonstrated a 50% or greater reduction (*n* = 6, responders) in Montgomery–Åsberg Depression Rating Scale (MADRS) scores from baseline to the 7th ketamine infusion during observational registry for safety and tolerability, and who were admitted as inpatients at the Department of Psychiatry, Medical University of Gdańsk (Poland)

^2^ Patients diagnosed with major depressive disorder who experienced less than 50% reduction (*n* = 22, non-responders) in Montgomery–Åsberg Depression Rating Scale (MADRS) scores from baseline to the 7th ketamine infusion during observational registry for safety and tolerability, and who were admitted as inpatients at the Department of Psychiatry, Medical University of Gdańsk (Poland)

^b^ denotes the use of the Wilcoxon test. Median scores (IQRs) are presented

## Discussion

This retrospective analysis indicates that sleep scores in inpatients receiving short-term intravenous ketamine treatment did not significantly improve, regardless of whether they were classified as responders or non-responders based on rater-based clinical measures. This study demonstrated a favorable safety and tolerability profile of short-term ketamine use in the TRD population.

This retrospective analysis with a primary focus on safety and tolerability is not in line with the current literature. Data on sleep changes during ketamine treatment in TRD are scarce with most findings reported in recent systematic reviews [24]. In prior clinical investigations involving 323 outpatient cases suffering from TRD in the context of MDD or bipolar depression, a treatment regimen consisting of four IV ketamine infusions was administered. Among these infusions, three were administered during the evening hours, while one was given in the morning or afternoon. Based on patient-reported outcomes (PRO), these interventions yielded notable enhancements in sleep, which, though modest in scale, made a meaningful contribution to the overall amelioration of depression severity, including a reduction in SI [16]. In another exploratory, post-hoc analysis of a randomized controlled trial involving 565 TRD within MDD patients with 343 who underwent treatment with esketamine nasal spray, noteworthy enhancements were observed in assessments conducted by evaluators compared to those receiving a placebo. These improvements were particularly pronounced on day 8 and continued to be significant in all subsequent assessments until day 28. Additionally, a higher proportion of patients treated with esketamine achieved clinically significant improvements in sleep, as defined by a reduction of at least two points in the score for item 4 of the MADRS compared to their baseline measurements [25]. A study enrolled 29 patients with TRD and utilized validated PROs, including the Pittsburgh Sleep Quality Index and Morningness-Eveningness Questionnaire. The investigation noted enhancements in sleep quality and a shift in the sleep phase towards earlier times, suggesting the potential chronotherapeutic properties associated with ketamine [26]. In a secondary analysis of 34 medication-free treatment-resistant MDD patients, with TRD defined as a failure to one antidepressant in the current depressive episode, underwent a single ketamine infusion followed by polysomnography. Notably, those who exhibited a decrease in nocturnal wakefulness during the timeframe spanning from midnight to 4:59 AM were more prone to experiencing a positive anti-suicidal response on the subsequent day [27]. Further evidence suggests a single IV ketamine infusion in TRD patients is correlated with an elevation in slow wave activity as measured objectively. Simultaneously, there are observable alterations in the levels of brain-derived neurotrophic factor, a recognized biomarker associated with neuroplasticity, which is believed to underlie the antidepressant effects of ketamine. Significantly, these observed changes exhibited a direct relationship, specifically in patients who demonstrated an amelioration in the severity of their depression. This observation suggests the involvement of sleep-related mechanisms as contributing factors in these neurobiological shifts [28]. Lastly, a case report in which a 55-year-old female with a history of depression and anxiety and four previous treatment failures reported improvement in sleep defined as a decrease from 11 to 7 on the Insomnia Severity Scale and a reduction from 13 to 8 on the Pittsburgh Sleep Quality Index [29]. Therefore, our results are somewhat unexpected. With recent conflicting results in the realm of sleep patterns in bipolar depression [30], to the best of our knowledge, there are no findings reporting a lack of ketamine’s sleep-promoting effect in MDD.

Since most of our research sample consists of non-responders, it may be hypothesized that ketamine does not exert sleep changes that are domain-specific. Despite a significant reduction observed in total depression scores, responders did not experience any meaningful sleep-related changes. The findings may deviate from existing literature owing to variations in study design. Unlike other studies that enrolled outpatients [16], participants in this investigation were hospitalized and mandated to remain in the hospital for the duration of the study. This discrepancy suggests that individuals in this study might have been more severely afflicted, necessitating hospital admission. Conversely, sleep alterations were assessed via self-report measures in this study, whereas alternative studies utilized clinician-rated assessments [25]. Another explanation for the observed results could be attributed to the use of the IDS-SR, contrasting with previous studies that utilized PROs specifically focused on sleep parameters [26]. An alternative interpretation for this observation may stem from the notably smaller sample size, indicating that the impact of ketamine on sleep could be relatively modest and detectable in larger cohorts. It should be noted that the sample size was limited, and only major differences could have been captured. The sample size wasn’t high enough to capture small but consistent changes as statistically significant.

This study has notable limitations. The primary limitation of this study is its small sample size, which may hinder its ability to detect statistically significant effects. The existing literature, although limited, indicates that the effect sizes for the sleep changes are generally small. Consequently, the study may be underpowered, making it difficult to definitively rule out alternative results. Furthermore, the study’s retrospective design means it was not initially intended to investigate sleep changes, potentially impacting the reliability of the findings. Thirdly, it lacked a placebo control group, randomization, or blinding, limiting generalizability to naturalistic observations. Fourthly, outcomes focused on short-term drug use with a mere 7-day follow-up, possibly missing long-term effects. Fifthly, sleep assessments relied solely on four items in the IDS SR-30 questionnaire, potentially missing out on the full scope of sleep disturbances. Lastly, objective sleep measures like actigraphy or polysomnography were absent, relying solely on patient-reported data, which precludes the examination of electrophysiological sleep parameters. Considering all limitations, it is advisable to approach the interpretation of the results with caution.

Nonetheless, this study possesses notable strengths. Patients were classified as responders and non-responders based on the widely accepted standard criteria for primary outcome measures, which aligns with both regulatory and academic consensus. Furthermore, PROs were systematically collected, and response patterns in patient-reported outcome measures were meticulously observed and analyzed by formal rater-based treatment outcome measures.

In summary, this study found no significant effect of ketamine on sleep in TRD. As the first study to report these negative results, contrasting with previous studies showing small effect sizes, the findings suggest that ketamine’s impact on sleep may be, in fact, minimal. Variations in study designs and populations (e.g., sample size, number of infusions, inpatient vs. outpatient) could account for these discrepancies. Therefore, ketamine may not reliably alleviate sleep disturbances, and an individualized treatment approach should be considered for managing sleep complaints. While minor changes may provide some benefit to patients, only significant differences are likely to influence clinical practice.

Future research endeavors should seek to provide further insight into sleep-related properties of ketamine. Preferably, randomized controlled trials should include primary outcome measures involving changes in sleep patterns. Additionally, given that ketamine was administered exclusively intravenously in this study, it may be worthwhile to explore whether other routes of administration, e.g., oral or subcutaneous affect sleep in TRD in a different manner.

## Conclusion and study limitations

This study adds to the existing body of literature concerning sleep-related alterations during ketamine therapy. Regardless of formal treatment outcome (classified as responder/non-responder), neither group demonstrated substantial changes in sleep quality in PRO measures. This report should be analyzed with caution primarily due to the modest sample size. The findings of this study scrutinizing a sample from a naturalistic registry support the safety and tolerability of ketamine administration in individuals with TRD.

## Data Availability

The datasets generated during and/or analyzed during the current study are not publicly available but are available from the corresponding author on reasonable request.

## Abbreviations

AMPAR: α-amino-3-hydroxy-5-methyl-4-isoxazolepropionic acid receptors
BDNF: brain-derived neurotrophic factor
DSM-5: Diagnostic and Statistical Manual of Mental Disorders
GABA: γ-aminobutyric acid
IDS SR-30: Inventory of Depressive Symptomatology 30-item
IV: intravenous
MADRS: Montgomery-Åsberg Depression Rating Scale
MDD: major depressive disorder
NMDAR: N-methyl-D-aspartate receptor
PRO: patient-reported outcome
RDoC: Research Domain Criteria
TRD: treatment-resistant depression
